# Rare Genomic Variants Link Bipolar Disorder with Anxiety Disorders to CREB-Regulated Intracellular Signaling Pathways

**DOI:** 10.3389/fpsyt.2013.00154

**Published:** 2013-11-28

**Authors:** Berit Kerner, Aliz R. Rao, Bryce Christensen, Sugandha Dandekar, Michael Yourshaw, Stanley F. Nelson

**Affiliations:** ^1^Semel Institute for Neuroscience and Human Behavior, University of California Los Angeles, Los Angeles, CA, USA; ^2^Department of Human Genetics, University of California Los Angeles, Los Angeles, CA, USA; ^3^Golden Helix, Bozeman, MT, USA; ^4^Department of Psychiatry and Biobehavioral Sciences, David Geffen School of Medicine, University of California Los Angeles, Los Angeles, CA, USA; ^5^Department of Pathology and Laboratory Medicine, University of California Los Angeles, Los Angeles, CA, USA

**Keywords:** bipolar disorder, exome sequencing, genetic risk factors, rare-variant common-disease model, ERK/MAPK and CREB-regulated intracellular signaling pathway, stress response, neuronal plasticity, threshold disease model

## Abstract

Bipolar disorder is a common, complex, and severe psychiatric disorder with cyclical disturbances of mood and a high suicide rate. Here, we describe a family with four siblings, three affected females and one unaffected male. The disease course was characterized by early-onset bipolar disorder and co-morbid anxiety spectrum disorders that followed the onset of bipolar disorder. Genetic risk factors were suggested by the early onset of the disease, the severe disease course, including multiple suicide attempts, and lack of adverse prenatal or early life events. In particular, drug and alcohol abuse did not contribute to the disease onset. Exome sequencing identified very rare, heterozygous, and likely protein-damaging variants in eight brain-expressed genes: IQUB, JMJD1C, GADD45A, GOLGB1, PLSCR5, VRK2, MESDC2, and FGGY. The variants were shared among all three affected family members but absent in the unaffected sibling and in more than 200 controls. The genes encode proteins with significant regulatory roles in the ERK/MAPK and CREB-regulated intracellular signaling pathways. These pathways are central to neuronal and synaptic plasticity, cognition, affect regulation and response to chronic stress. In addition, proteins in these pathways are the target of commonly used mood-stabilizing drugs, such as tricyclic antidepressants, lithium, and valproic acid. The combination of multiple rare, damaging mutations in these central pathways could lead to reduced resilience and increased vulnerability to stressful life events. Our results support a new model for psychiatric disorders, in which multiple rare, damaging mutations in genes functionally related to a common signaling pathway contribute to the manifestation of bipolar disorder.

## Introduction

Bipolar disorder is a common, severe psychiatric disorder with onset in adolescence and early adulthood. Broadly defined, the disease affects about 2% of the world’s adult population (1). At the core of the disorder are recurrent and severe disturbances of mood, which cycle between mania and depression. In addition, severely affected individuals often develop co-morbid psychiatric disorders, including eating disorders, anxiety disorders, and addictions (2). Stressful life events could trigger the disease onset (3, 4). However, familial aggregation has indicated a genetic predisposition and twin studies have supported this hypothesis (5). Nevertheless, no major disease-causing genetic variant has been identified despite enormous efforts involving linkage analysis in multi-generation families and association analysis in large population samples. Therefore, most of the genetic risk remains unexplained. So far, the data neither support a disease model which favors a single rare mutation with large effect, nor a disease model based on common variants with small effects. As a consequence, the patho-mechanisms of this common complex disorder remain elusive (6–9).

The question remains how genetic risk factors contribute to the manifestation of bipolar disorder. If we could answer this question, early intervention and effective treatment could become a reality. Here, we propose a disease model in which multiple very rare, damaging variants increase the vulnerability to adverse life events. Variants could contribute jointly to a disease phenotype, if they affect a common pathway. According to our current knowledge, most disease-causing mutations are rare. Disease-causing mutations are often found in protein coding regions and they have deleterious consequences for the structure and function of the encoded proteins. Rare disease-causing mutations in families with Mendelian disorders have been identified through exome sequencing (10). But also in common complex disorders, researchers have begun to apply this method (11). In epilepsy, investigators discovered rare and potentially deleterious missense mutations in known disease-causing genes. While these mutations were present in cases as well as in controls, the combination of multiple deleterious mutations was unique to the cases (12, 13). Exome sequencing in schizophrenia revealed a large number of very rare and *de novo* mutations that were present in cases only and absent in controls supporting a contribution to disease risk (14). Exome sequencing in families offers an advantage over case-control studies for the identification of rare variants. Because disease alleles are shared identity-by-descent among multiple affected family members, segregation analysis could limit the number of alleles to be considered. While the results of exome sequencing in common complex disorders support a “rare-variant common-disease” model, they also indicate a high degree of pathophysiological heterogeneity. Pathophysiological heterogeneity implies that genetic risk factors should be shared among all affected individuals within one family, but genetic risk variants might differ between families. Rare variants could then be further evaluated with well powered case/control studies and functionally assessed to confirm the identified candidate genes.

**Figure 1:**
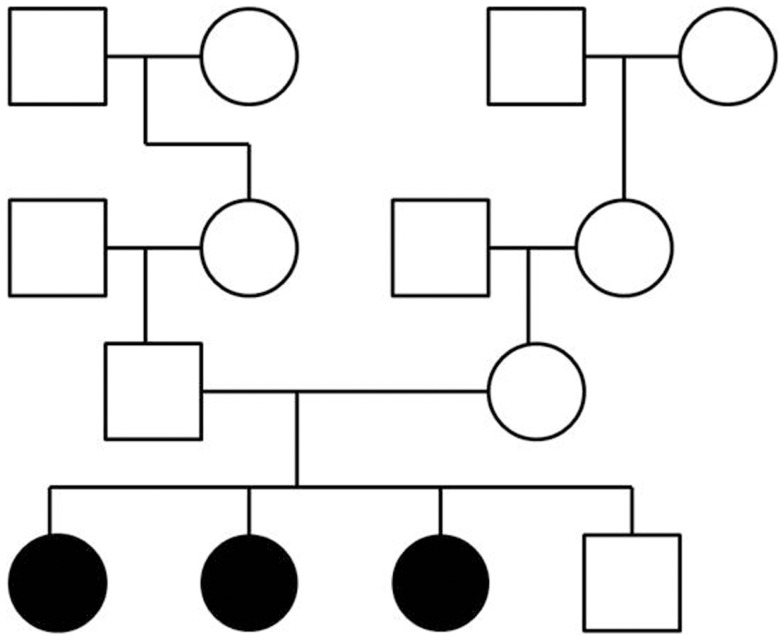
**Pedigree of a family with three siblings diagnosed with bipolar disorder and co-morbid anxiety spectrum disorders**. The three-generation pedigree did not indicate additional psychiatric disorders on the father’s side or the mother’s side of the family. In this pedigree, a circle indicates a female, a square indicates a male. A filled symbol indicates a person affected with bipolar disorder and co-morbid anxiety spectrum disorders. An unfilled symbol indicates a healthy individual. Family relationships are indicated by lines.

To perform exome sequencing in bipolar disorder patients, we selected a family with three affected sisters and an unaffected brother (Figure 1). Both parents were healthy and the family history had been unremarkable for psychiatric disorders over four generations. This mode of disease transmission initially appeared to be autosomal recessive Mendelian inheritance. We sequenced the four siblings exome-wide to identify rare coding, protein-damaging, and potentially causal mutations that were shared by the affected siblings. In addition, we used two sets of healthy controls to distinguish non-pathogenic variants from potentially disease-causing variants. The first control data set consisted of a smaller number of individuals from the same ethnic background; the second set was a large and ethnically diverse control sample. All control DNAs were processed under identical capture and sequencing conditions.

## Materials and Methods

### Sample

The Caucasian family was recruited as part of the National Institute of Mental Health (NIMH) Human Genetics Initiative (15). Information about the disease course and disease symptoms of the family members had been ascertained with the Diagnostic Instrument for Genetic Studies (DIGS, Version 4). The disease history had been evaluated with the Family Interview for Genetic Studies (FIGS) (16). To protect the privacy of the family members, only summary information about disease symptoms and disease course will be given here. Further details can be obtained from the investigators upon request. The phenotype of the three affected family members was consistent with the diagnosis of bipolar disorder. Both parents were reportedly healthy, and the family history was unremarkable in terms of psychiatric disorders over at least three generations. At the time of the interview, the affected family members had been ill for more than 20 years. The age of onset, the symptoms, the number of episodes, and even the co-morbidity conditions were remarkably similar in all three affected females. Symptoms of depression manifested between the ages of 14 and 17 as the first reported sign of a psychiatric disorder. Stressful life events appeared to have triggered the onset of depression in two of the patients. There was no history of alcohol or illegal substance abuse at that time. During the entire disease course, severe depression was the predominant symptom, leading to several suicide attempts with hospitalization. Symptoms of depression were first treated with tricyclic antidepressants and then with selective serotonin reuptake inhibitors (SSRIs). Several years after the onset of depression, during late adolescence and early adulthood, all three sisters manifested symptoms of mania accompanied by brief psychotic episodes with auditory hallucinations, visual hallucinations, and delusions. Psychotic symptoms were treated with chlorpromazine. Lithium was added to prevent recurrence of mania. One individual was also treated with the anticonvulsant Lamotrigine. Mood symptoms were triggered by hormonal changes, especially during pregnancy and during the postpartum period. All three affected individuals developed social phobia and panic disorder. In addition, one of the three siblings developed rapid-cycling and mixed-cycling bipolar disorder. She was also diagnosed with learning disability, attention-deficit hyperactivity disorder (ADHD), obsessive compulsive disorder (OCD), and eating disorder (anorexia nervosa), but symptoms of these disorders were not present in the other siblings. Despite several hospitalizations for severe depression, none of the siblings achieved complete remission. Nevertheless, all three patients lived in stable social relationships. Two of the sisters finished college, married, and had children. The male sibling was healthy and did not have any psychiatric symptoms.

### Exome capture and re-sequencing

Exome capture and DNA sequencing followed a standardized protocol. We sequenced the exomes of all three affected females and the unaffected male. DNA of the parents was not available. We isolated genomic DNA from immortalized lymphoblastoid cell lines following standard protocols. After DNA quality control using the Qubit Fluorimeter (Invitrogen) and the Bioanalyzer (Agilent), exome capture was performed with the Illumina TruSeq™ Exome Enrichment Kit according to the manufacturer’s protocol. This assay is designed to target 201,121 exons in 20,794 genes (based on the NCBI37/hg19 reference genome) covering about 97% of the CCDS coding exons and 96% of the RefSeq coding exons with uniform coverage across 62 Mb. We constructed the Illumina paired-end sequencing libraries with the TruSeq DNA sample preparation kit according to Illumina protocols (Illumina Inc., San Diego, CA, USA). Samples were sequenced on the Illumina HiSeq 2000, to generate a total of 52.3 million 100 bp paired-end reads per sample. Base-calling was performed with real-time analysis (RTA) software from Illumina. We also processed 53 Caucasian control exomes under the same capture and sequencing conditions, and these exomes were included in the base-calling and variant-calling process. In addition, we used an internal exome data set of more than 200 individuals sequenced for other medical conditions as further controls. This second set of controls reflected California’s ethnically diverse population and was used to guard against selecting ethnic-specific variants.

### Sequence alignment, variant calling, and quality control

Sequencing reads were aligned to the reference genome (human_g1k_v37.fasta) using the Novoalign software package. The software SAM Tools (Version 0.1.15) was used to sort the aligned BAM files. Variants were called simultaneously for cases and controls using the Genome Analysis Toolkit (GATK) Unified Genotyper tool, which was run in multiple-sample mode according to published best practice recommendations. Potential PCR duplicates were removed with Picard. On average, 90.0% reads aligned uniquely to the reference genome. The PCR duplication rate varied between 6.9 and 24.0%, and there was an average estimated library size of 222 million unique fragments. The on-target rate, or capture specificity, varied from 89.2 to 89.9%. The mean coverage across the captured regions was 52.84, and approximately 87.5% of the targeted bases were covered by ≥10 reads for each exome. We inspected all regions of interest manually for correct alignment.

We confirmed relationships by running PLINK to calculate a matrix of genome-wide average identity-by-state (IBS) pair-wise identities. After removing synonymous variants and low quality calls, we focused on those variants that were shared by the affected family members. The prediction tools Sorting Intolerant From Tolerant (SIFT), Polymorphism Phenotyping Version 2 (PolyPhen-2), MutationTaster, and MutationAssessor were used to evaluate potentially damaging consequences of the variants (17–21). While all these functional predictors use similar information to predict the potential disease-causing effects of genomic variants, there are significant differences in the weighting of the different pieces of information, which could lead to discrepancies in the conclusions. GERP++ RS and PhyloP were used to determine the strength of past purifying selection and the conservation scores of the mutated gene regions (22). The functional prediction algorithms were available through the SVS interface from Golden Helix and dbNSFP as the intermediate source (23).

The genes with filtered variants were examined for evidence of expression in brain using BioGPS and the Illumina Human Body Map. The Ensembl database, online Mendelian inheritance in man (OMIM), the Mouse Genome Informatics (MGI) website, Human Mutation Data Base (HMDB), and PubMed were used to further characterize the selected variants. We manually determined the location of the variants in relation to functional regions in the gene sequence or protein structure. Potential associations with a behavioral phenotype in animal models, as well as *in vitro* or *in vivo* studies in humans, were examined by searching the published literature. The list of selected genes was then examined for the presence of equally or more damaging mutations in the controls.

### Copy number variation analysis

We examined the presence or absence of copy number variations (CNVs) that were shared among the affected family members using the CNV-calling algorithm for exome sequencing eXome-Hidden Markov Model (XHMM) (24). We ran XHMM on the four family members together with 15 additional bipolar disorder samples and 55 healthy controls using default settings. Next, we filtered CNVs by quality score as previously described, and eliminated CNVs in which the probability of a CNV existing in the region was <0.60. We also excluded deletions or duplications for which the affected family members were discordant. Finally, we searched DECIPHER to determine the population frequency and previously known disease associations of the remaining CNVs. DECIPHER is a CNV database for clinically significant structural variants, which incorporates also a series of normal CNV datasets, including the 1000 Genomes Project and other published sources (25).

### Confirmation of variants

We confirmed the presence of the selected variants with SNPtype™ Assays from Fluidigm, or with Taqman 3 according to the company’s protocols, if the genomic regions did not exceed the maximum level of GC content allowed.

## Results

Our analysis revealed a total of 432,621 variants present in cases and controls combined. Only 33,569 variants were present in at least one of the four exomes, and no more than 17,116 variants were considered non-synonymous changes. There was no evidence for shared CNVs in this family. First, we searched for shared homozygous protein-damaging mutations, because the inheritance pattern indicated genetic risk factors that follow a recessive mode of inheritance. We identified 46 homozygous variants that were shared by all three affected family members and absent in the unaffected brother. All these variants were also present in the homozygous state in multiple controls. Only 37 of these variants were considered to be potentially damaging by at least one of the variant effect predictors, but none of these polymorphisms were predicted to be damaging by at least three predictors. Therefore, a pathogenic role in bipolar disorder was highly unlikely. In addition, no gene carried two damaging mutations that were shared by the affected siblings. This excluded compound heterozygous mutations. Thus, we concluded that our data did not support a classic recessive mode of inheritance for bipolar disorder in this family.

Next, we focused on heterozygous variants that were present in affected individuals only and not present in the unaffected brother or the controls. We found 326 variants in which the minor allele was shared by all affected family members. Only 20 variants were predicted to be potentially damaging by at least one of the four predictors. Not more than eight mutations were strongly predicted to be damaging by at least three predictors or not predicted to be tolerated by more than one predictor (Table 1). These variants consisted of three previously known but extremely rare variants. The other five mutations were novel compared to dbSNP (last accessed on 7/31/2013). All mutated genes were expressed in brain. The variant-carrying genes were IQUB (IQ motif and ubiquitin domain containing), JMJD1C (jumonji domain containing 1C), GADD45A (growth arrest and DNA-damage-inducible, alpha), GOLGB1 (golgin B1), PLSCR5 (phospholipid scramblase family, member 5), VRK2 (vaccinia related kinase 2), MESDC2 (mesoderm development candidate 2), and FGGY (FGGY carbohydrate kinase domain containing). All genes are expressed in brain and highly conserved, according to the Ensembl data base, GERP++ RS, and PhyloP scores (26, 27).

**Table 1:** **In this table, we have summarized information on shared genomic variants in a family, in which three siblings were affected with bipolar disorder and anxiety spectrum disorders**.

**(A) Potentially damaging variants in nine brain-expressed genes were shared by the affected family members**.						
Location	Gene	Transcript	Exon	Coding	Protein	Zygosity
7:123109272	IQUB	NM_178827	9	c.1577T > A	p.Val526Glu	het
10:64967788	JMJD1C	NM_032776	10	c.3641A > G	p.His1214Arg	het
1:68152073	GADD45A	NM_001199741	2	c.85G > C	p.Glu29Gln	het
3:121435874	GOLGB1	NM_004487	9	c.983T > C	p.Val328Ala	het
3:146311810	PLSCR5	NM_001085420	4	c.350G > A	p.Arg117Gln	het
3:145917659	PLSCR4	NM_020353	6	c.565A > G	p.Met189Val	het
2:58315517	VRK2	NM_001130480	6	c.386A > G	p.Gln129Arg	het
15:81274319	MESDC2	NM_015154	2	c.418C > T	p.Leu140Phe	het
1:60019796	FGGY	NM_001113411	8	c.800G > T	p.Gly267Val	het

**(B) Quality control measures for the rare variants indicated high quality reads and good coverage**.					
Location	Gene	Identifier	Quality score	Filter result	Average depth
7:123109272	IQUB	novel	1148.24	Pass	68.1677
10:64967788	JMJD1C	novel	3935.47	Pass	141.0839
1:68152073	GADD45A	novel	747.91	Pass	27.9346
3:121435874	GOLGB1	rs140932474	4257.63	Pass	125.7677
3:146311810	PLSCR5	rs199965523	4061.42	Pass	137.5935
3:145917659	PLSCR4	rs139054640	1708.17	Pass	68.9742
2:58315517	VRK2	novel	3801.98	Pass	126.9355
15:81274319	MESDC2	novel	1624.88	Pass	68.271
1:60019796	FGGY	rs142088608	2029.22	Pass	83.0387

**(C) Functional prediction algorithms indicate deleterious effects of the shared variants on protein structure and function**.							
Location	Gene	SIFT	SIFT prediction	PolyPhen-2	PolyPhen-2 Prediction	MutationTaster	MutationTaster prediction
7:123109272	IQUB	0	Damaging	0.998	Probably damaging	0.641624	Disease-causing
10:64967788	JMJD1C	0.04	Damaging	0.504	Possibly damaging	0.614459	Disease-causing
1:68152073	GADD45A	0.03	Damaging	0.817	Possibly damaging	0.384507	Polymorphism
3:121435874	GOLGB1	2	?	−1	?	−1	?
3:146311810	PLSCR5	0	Damaging	0.999	Probably damaging	−1	?
3:145917659	PLSCR4	0.25	Tolerated	0.542	Possibly damaging	0.283731	Polymorphism
2:58315517	VRK2	0.04	Damaging	0.631	Possibly damaging	0.916062	Disease-causing
15:81274319	MESDC2	0	Damaging	1	Probably damaging	0.999964	Disease-causing
1:60019796	FGGY	2	?	0.999	Probably damaging	0.999565	Disease-causing

Location	Gene	MutationAssessor	MutationAssessor prediction	GERP++ RS	PhyloP
7:123109272	IQUB	3.16	Predicted functional (medium)	5.51	2.105
10:64967788	JMJD1C	1.79	Predicted non-functional (low)	5.58	2.114
1:68152073	GADD45A	2.265	Predicted functional (medium)	4.58	1.324
3:121435874	GOLGB1	2.015	Predicted functional (medium)	4.54	2.33
3:146311810	PLSCR5	3.82	Predicted functional (high)	5.69	2.679
3:145917659	PLSCR4	0.58	Predicted non-functional (neutral)	3.69	0.971
2:58315517	VRK2	2.32	Predicted functional (medium)	5.63	2.281
15:81274319	MESDC2	2.555	Predicted functional (medium)	5.32	2.487
1:60019796	FGGY	3.425	Predicted functional (medium)	5.28	2.736

(A) describes the exact chromosomal location of the identified variant for each gene, followed by the identification number of the major gene transcript. For each variant, we identified the exon, in which the mutation was found, as well as the resulting change in the coding sequence of the gene and the amino acid change in the protein. We also indicate if the change occurred on either both alleles (homozygous) or only on one of the alleles (heterozygous).

(B) indicates if the genomic variant in the specific gene is a novel occurrence or has been described before in genomic data bases, as indicated by the rs identification number. The quality score indicates the overall quality of the sequencing reaction according to GATK. Filter results indicate if a variant met pre-specified quality control thresholds. The table also indicates the average read depth of the sequencing reaction for a particular base pair.

(C) summarizes the results of the functional prediction algorithms SIFT, PolyPhen-2, MutationTaster, and MutationAssessor. Both the scores and the interpretation are given. It also indicates the degree of evolutionary constraint at the base pair location predicted by the GERP++ RS and PhyloP scoring systems (26, 27). While not all functional predictors agree with the deleterious consequences of the base pair change, a clear trend is obvious. Functional experiments are recommended to confirm the predictions.

A ? indicates that the prediction is unknown.

The variants were found in the genes IQUB, IQ motif and ubiquitin domain containing; JMJD1C, jumonji domain containing 1C; GADD45A, growth arrest and DNA-damage-inducible, alpha; GOLGB1, golgin B1; PLSCR5, phospholipid scramblase family, member 5; PLSCR4, phospholipid scramblase family, member 4; VRK2, vaccinia related kinase 2; MESDC2, mesoderm development candidate 2; FGGY, carbohydrate kinase domain containing.

IQUB is essential for the recycling of at least two membrane-bound receptors, HTR6 (5-hydroxytryptamine [serotonin] receptor 6) and melanin-concentrating hormone receptor 1 (MCHR1) (28). Both receptors are coupled to G-proteins and they transmit their signal through MAP kinases. IQUB is located on chromosome 7q31.32. The novel mutation in Exon 9 leads to an amino acid change from valine to glutamine (c.1577T > A, p.Val526Glu). Mutations in this codon result in nonsense-mediated decay. All four functional predictors consider the variant deleterious for the protein structure and function. We did not find equally damaging mutations, even in other regions of the gene, in more than 200 healthy controls. While the sequencing results received high quality scores, it was challenging to confirm the variant with genotyping, possibly due to the high GC content of the gene region.

The histone demethylase JMJD1C, also known as TRIP8 (thyroid hormone receptor beta [TR beta]-binding protein 8) is involved in hormone-dependent transcriptional regulation. The protein is a co-factor of several DNA-binding hormone receptors, including androgen receptor (AR), thyroid hormone receptor beta (THRB), and retinoid X receptor, alpha (RXRA) (29–32). JMJD1C is involved in ERK/MAPK and CREB-regulated intracellular signaling pathways, where it directly interacts with GADD45A (growth arrest and DNA-damage-inducible, alpha) (33). The gene has been mapped to chromosome 10q21.3. The identified novel variant causes an amino acid change from histidine to arginine in Exon 10 of the gene (c.3641A > G, p.His1214Arg). While three predictors consider the variant deleterious, MutationAssessor predicts only low functionality. We did not find any equally damaging mutation in this gene in the controls.

GADD45A is another protein in the ERK/MAPK and CREB-regulated intracellular signaling pathways. It directly stimulates MEKK4 (mitogen-activated protein kinase kinase kinase 4) activity. It is also an antagonist of GSK3beta (glycogen synthase kinase 3 beta), which inhibits MEKK4 kinase (34, 35). GADD45A is located on chromosome 1p31.2. The novel mutation in Exon 2 (c.85G > C [p.Glu29Gln]) affects the protein kinase-regulating region of the protein. While three predictors consider the variant damaging, MutationTaster labels it a polymorphism. We did not find any deleterious or novel mutation in this gene in the controls.

GOLGB1, also known as Giantin, is a brain-expressed gene and an integral part of the ERK/MAPK and CREB-regulated intracellular signaling pathways (36). The protein is associated with the Golgi apparatus and participates in G-protein coupled receptor recycling. One of these receptors is the adrenoceptor alpha 2B (ADRA2B), a key player in the regulation of neurotransmitter release in the central nervous system (37). The gene is located on chromosome 3q13. The known variant (rs140932474) is found in Exon 9 and results in an amino acid change from valine to alanine. The variant affects a glutamine-rich functional region of the protein, but only the MutationAssessor considers the change to be functional. All other predictors do not provide any information about functional consequences of the variant. The same variant segregated with symptoms of depression in a second family with bipolar disorder in our data set. The Minor Allele Frequency (MAF) of this variant in the general population is unknown.

PLSCR5 belongs to the scramblase protein family. Scramblase proteins are membrane-associated proteins with transcription factor activity. They play a prominent role in G-protein coupled receptor signaling and gene expression regulation. It is likely that they interact with Phospholipase C (PLC), a central protein in the ERK/MAPK and CREB-regulated intracellular signaling pathways (38–40). PLSCR5 is located on chromosome 3q24 (3:146311810). We identified the known variant rs199965523 in this gene. Three predictors consider the variant damaging. The base change results in a stop-gain through a transversion from G to A in Exon 4 of the gene (c.350G > A [p.Arg117Gln]). The mutated gene region encodes a tubby C-terminal-like domain, which is predicted to be a binding site for Phospholipase C. Rs199965523 has been observed only twice before, once in the 1000 Genomes Project in a Japanese patient and again in the NHLBI GO Exome Sequencing Project. We don’t know if individuals carrying this mutation were healthy. The identified mutation was absent in our controls, but we found a potentially equally damaging mutation in the vicinity of rs199965523 in five healthy individuals.

PLSCR4 and PLSCR5 are the only brain-expressed genes in the scramblase protein family and their expression pattern suggests an important role in prenatal brain development and brain function throughout the life span (41, 42). Therefore, it might be important that we found another known variant (rs139054640) in the gene PLSCR4 (phospholipid scramblase family, member 4). The gene is located in close proximity to PLSCR5 on chromosome 3q24. The variant is in Exon 6 of the gene and it has been described before, twice in the ESP6500:European_American project and once in the NHLBI-ESP:ESP_Cohort_Populations. The mutation is located in the characteristic scramblase region shared with PLSCR5, but the protein does not appear to have the tubby-like function. Only PolyPhen-2 considers the variant to be potentially damaging. Therefore, the variant rs139054640 did not pass our filtering criteria. In addition, the controls had equally damaging mutations in other gene regions. However, only the cases carried the unique combination of the two variants in the scramblase genes. Even though rs139054640 is less likely to be pathogenic, we cannot exclude potentially damaging consequences of the combined effect of the two mutations.

VRK2 is a serine/threonine protein kinase and a modulator of the ERK/MAPK and CREB-regulated intracellular signaling pathways (43). Activation of VRK2 results in reduced phosphorylation of key proteins in this pathway and reduced MEK-induced gene transcription (44, 45). The novel gene variant leads to an amino acid change in the serine/threonine double-specific kinase domain of the protein. All four predictors consider the variant damaging. While this variant was present only in the affected family members, a previously known variant (rs144870539) was present in the serine/threonine protein kinase domain in two controls. Rs144870539 has a MAF of 0.003 in European populations.

MESDC2 is a chaperone in the Wnt/β-catenin-signaling pathway, which contributes to neuronal plasticity jointly with the ERK/MAPK and CREB-regulated intracellular signaling pathways (46–48). The protein is also essential for establishing polarity during embryonic development and for mesoderm induction (49). The novel mutation is in Exon 2 of the gene and affects the functional domain of the protein. While the mutation was present only in cases, another known and potentially damaging variant was also present in the same functional region in the controls.

FGGY is a phosphotransferase and a member of the FGGY kinase family (50). The gene is expressed in brain. The shared variant is a known polymorphism (rs142088608), which has been described only once before in the European sample of the 1000 Genomes Project. However, several potentially damaging mutations in the vicinity of this variant were present in the controls.

**Figure 2:**
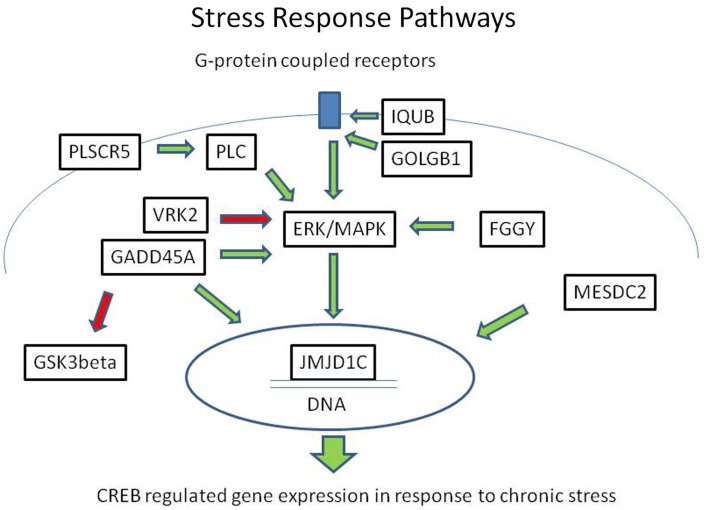
**Mutations in the ERK/MAPK signaling pathway and related second messenger systems**. The ERK/MAPK signaling pathway and related second messenger systems play a central role in neuronal and synaptic plasticity, affect regulation, and response to chronic stress. Mutations in genes related to these pathways could lead to reduced resilience and increased vulnerability. In a family with bipolar disorder and co-morbid anxiety spectrum disorders, we have identified potentially damaging mutations in genes related to these pathways. The genes encode proteins that are key regulators at several levels, from the cell membrane to the nucleus. IQUB and GOLGB1 are involved in G-protein coupled receptor recycling. FGGY, VRK2, GADD45A, and PLSCR proteins influence signal transduction through the ERK/MAPK messenger cascade. MESDC2 and JMJD1C influence CREB-regulated gene expression in the nucleus. Direct physical interaction has been reported between GADD45A and JMJD1C. These second messenger systems are also the target of lithium and valproic acid, which are commonly used to treat bipolar disorder. Green arrows indicate activating influences and red arrows indicate inhibiting influences.

In summary, we identified eight rare, protein-damaging mutations in eight brain-expressed genes that were shared by three siblings affected with bipolar disorder. These mutations consisted of five novel sequence changes and three previously reported very rare variants. The genes encode proteins that modulate ERK/MAPK and CREB-regulated intracellular signaling pathways at several levels, from membrane-bound receptor recycling to gene expression regulation (Figure 2). While equally damaging mutations were also present in some of these genes in the controls, the combination of the variants was unique to the cases.

## Discussion

Strong heritability of bipolar disorder has been supported by many studies, but the identification of causal variants has been challenging. Common genomic variants have been explored with genome-wide association studies in large population samples. These studies might have identified some common markers indicating slightly increased risk, but so far they have not revealed a specific disease-causing mutation that could provide insight into disease mechanisms. Recently, exome-wide sequencing studies have suggested rare causal variants and even combinations of multiple rare, damaging variants with small to moderate effect as risk factors for psychiatric disorders. To find further support for this “Multiple-Rare-Variants Common-Disease Model,” we sequenced the exomes of affected and unaffected family members in a small pedigree with bipolar disorder and examined all potentially damaging mutations that were shared by the affected family members.

The pedigree suggested an autosomal recessive mode of inheritance in this family. The parents had been healthy, but multiple children had been diagnosed with bipolar disorder in the absence of adverse life events or substance abuse. Nevertheless, exome-wide sequencing failed to identify a rare, homozygous, damaging mutation, or compound heterozygous mutations in the same gene that could explain this pattern of disease aggregation. Therefore, we did not find support for a recessive disease model in the sequence data. Alternatively, one of the parents could have carried a *de novo* germ-line mutation with large and highly penetrant effect. In this case, the mutation would not be present in the parent’s DNA and the parent would be unaffected. But the mutation could be transmitted through the germ cells to the children, who would manifest the disease. To support this hypothesis, further studies would have to demonstrate the absence of the mutation in the DNA of both parents and a damaging effect in functional assays. Unfortunately, parental DNA was not available to confirm this hypothesis. Alternatively, the disease could be caused by a combination of multiple rare, damaging mutations in multiple genes that function in the same intracellular signaling pathway. Under this disease model, each parent would share only a fraction of the mutations with the affected siblings, but a normal phenotype could result, because compensatory mechanisms would prevent the disease manifestation indicating robustness of the intracellular signaling cascades. This disease model resembles the threshold disease model well known in cancer genetics. Under this model, multiple mutations lead up to a threshold, beyond which disease manifestations become likely. The combination of mutations could weaken central intracellular signaling pathways resulting in reduced resilience and increased vulnerability to disease.

Our results support the idea that very rare mutations play a potentially pathogenic role in bipolar disorder. In our exploratory study, we identified eight very rare, damaging mutations shared by three siblings affected with bipolar disorder and anxiety spectrum disorders. Further evidence supported a potential causal relationship to these disorders. First, all identified mutations were very rare in healthy individuals. In fact, five of these mutations were novel, and three had been described before in only one or two individuals worldwide. Second, most functional predictors agreed on the damaging consequences of the mutations. Third, the four genes did not carry equally or more damaging mutations in the controls. This fact confirmed the highly conserved nature of the genes. Still, we were able to confirm the segregation of one of these variants (rs140932474 in the gene GOLGB1) with symptoms of depression in a second bipolar family sequenced in our laboratory. Last but not least, the shared mutations were in genes that are functionally related to the ERK/MAPK and CREB-regulated intracellular signaling pathways.

The ERK/MAPK and CREB-regulated intracellular signaling pathway is central to neuronal plasticity, affect regulation and response to chronic stress (51, 52). In mouse models, a close relationship has been demonstrated between exposure to chronic stress and hyper-phosphorylation in the extracellular signal-regulated kinase (ERK) pathway leading to depression (53). Hyper-activation in the ERK/MAPK signaling pathways resulted in long-term changes in multiple neurotransmitter systems and neuronal atrophy (54, 55). Animal models have substantiated the hypothesis that abnormalities in these pathways are responsible for affect dysregulations, such as anxiety and depression (56–59). If hyper-phosphorylation could lead to behavioral inhibition, as seen in depression, hypo-phosphorylation could result in the opposite behavior, as seen in mania. Therefore, it is easily perceivable that functional impairment in activating and inhibiting proteins in these pathways may result in imbalances that could lead to the cycling pattern of mood stages observed in bipolar disorder. Acute and chronic stress could overload an already vulnerable system that might have been balanced by compensatory mechanism. Commonly used mood-stabilizing medications, including lithium, valproic acid, and tricyclic antidepressants act on proteins in the ERK/MAPK signaling cascades and also regulate gene expression (60–63). For example, valproic acid up-regulates GADD45A, a gene identified in our study (64).

Further evidence is accumulating that genes identified in this study are involved in physiological processes that have been related to psychiatric disorders. GADD45A directly influenced neurite complexity during brain development and reduced neurite outgrowth in response to environmental stress (65–67). The expression of GADD45A is regulated by Period2, a key regulator of circadian rhythms (68). JMJD1C has been implicated in the pathophysiology of autism (69). In the mouse, the protein regulates the transition from prepubertal stages to puberty by replacing WHISTLE as regulator of steroid hormone synthesis (70). The protein could also be a link between serotonin and thyroid hormones, which might be relevant for the pathophysiology of mood disorders (71). In addition, mutations in this gene could lead to abnormal methylation (72). PLSCR4 was among a small number of genes with significantly reduced expression in the brain of suicide victims with bipolar disorder and schizophrenia compared to controls (73). These findings suggest a close connection between scramblase genes and the phenotype of depression, suicide attempts, and psychosis. Wnt/β-catenin signaling is essential for cellular resilience and neural plasticity (74). This signaling pathway is also a target of valproic acid. Therefore, it is likely that mutations in these genes could contribute to the pathophysiology of bipolar disorder and explain drug resistance in certain patients. Our report provides the first indication for specific, potentially disease-causing mutations in the ERK/MAPK and CREB-regulated intracellular signaling pathways.

While exome sequencing has identified many disease-causing mutations in Mendelian disorders, this approach has obvious limitations. First, exome sequencing is limited to the coding regions of the genome. This method does not capture variants in regulatory regions of genes, such as the promoter regions, the 3′region or intron regions with regulatory functions. Second, we did not address differences in expression levels of non-coding regulatory RNAs or the effect of methylation differences between affected and unaffected family members. These studies would be a valuable addition to the field. Third, our study explores only genomic variants in a single family, in which multiple members had been diagnosed with bipolar disorder. These families are very rare. While our results could provide insight into disease patho-mechanisms, it remains to be determined how the disease model can be generalized and translated to the far more common sporadic cases of bipolar disorder or anxiety spectrum disorders. Also, single-family studies have the potential to be vulnerable to confounding factors. The affected family shared a number of symptoms, including depression, suicide attempts, panic disorder, mania, and psychosis. It is unclear whether these symptoms characterize specific expressions of bipolar disorder or whether they are caused by independent genetic or environmental risk factors. Studies in larger samples ascertained for these co-morbid disorders will be necessary to exclude potential confounding factors.

In summary, we consider our contribution to be an exploratory evaluation of all coding variants shared by three siblings with bipolar disorder and anxiety spectrum disorders. Our results provide evidence for a “multiple-rare-variant common-disease model.” While the data are clearly insufficient to conclude causality of any single variant that we identified in this family or provide any statistical evidence for disease association, they provide preliminary data for larger studies designed to test the hypotheses that arose out of this exploration. In order to generalize our results, replication studies would need to confirm the disease association and functional studies would need to test causality. Nevertheless, we are convinced that it is highly valuable to share our results on the background of other larger ongoing sequencing efforts in bipolar disorder and other psychiatric disorders.

## Authors Contribution

Berit Kerner conceived the study, designed the experiment, analyzed and interpreted the data, and wrote the manuscript. Aliz R. Rao aligned the sequence reads, produced the VCF files, performed the CNV analysis and analyzed, and interpreted the CNV data. Bryce Christensen assisted with the bioinformatics analysis of the data using the SVS software. He contributed to writing and editing of the manuscript. Sugandha Dandekar confirmed the variants with SNP genotyping. Michael Yourshaw provided the sequencing data for the controls and assisted in the alignment of the sequencing reads and the creation of the VCF files. He also edited the paper. Stanley F. Nelson provided expertise and the facility for exome-wide sequencing. He supervised the sequencing analysis, contributed the control DNA, discussed the design of the study and the interpretation of the data, and edited the manuscript.

## Conflict of Interest Statement

The authors declare that the research was conducted in the absence of any commercial or financial relationships that could be construed as a potential conflict of interest.

## Web Resources

– **National Institute of Health Human Genetics Initiative**, *https://www.nimhgenetics.org/nimh_human_genetics_initiative*
– **BioGPS**, *http://biogps.org*
– **CCDS coding exons**, *http://www.ncbi.nlm.nih.gov/CCDS/CcdsBrowse.cgi*
– **dbNSFP**, *http://dbnsfp.houstonbioinformatics.org/dbNSFPzip/dbNSFP2.0b1_variant.readme.txt*; *https://sites.google.com/site/jpopgen/dbNSFP*
– **dbSNP**, *http://www.ncbi.nlm.nih.gov/projects/SNP*
– **DECIPHER**, *http://decipher.sanger.ac.uk*
– **Ensembl Genome Browser**, *http://ensembl.org*
– **XHMM (eXome-Hidden Markov Model)**, *http://atgu.mgh.harvard.edu/xhmm*
– **FATHMM**, *http://fathmm.biocompute.org.uk/*
– **Genome Analysis Toolkit (GATK) Unified Genotyper tool**, ftp://gsapubftp-anonymous@ftp.broadinstitute.org *http://www.Broadinstitute.org/gsa/wiki/index.php/Best_ Practice_Variant_Detection_withthe_GATK_v3*
– **GenomeRNAi**: *http://genomernai.dkfz.de/GenomeRNAi/newexternalexperimentdetails*
– **Genomic Evolutionary Rate Profiling (GERP)**, *http://mendel.stanford.edu/SidowLab/downloads/gerp*
– **Golden Helix**, *http://www.goldenhelix.com*
– **Human Gene Mutation Database**, *http://www.hgmd.cf.ac.uk/ac/index.php*
– **Illumina Human Bodymap**, *http://www.ensembl.info/blog/2011/05/24/human-bodymap-2-0-data-from-illumina*
– **Mouse Genome Informatics (MGI)**, *http://www.informatics.jax.org*
– **MutationAssessor**, *http://mutationassessor.org/*
– **MutationTaster**, *http://www.mutationtaster.org/StartQueryEngine.html*
– **NHLBI-ESP:ESP_Cohort_Populations**, *http://www.ensembl.org*
– **Novocraft Short Read Alignment Package**, *http://www.novocraft.com*
– **Online Mendelian Inheritance in Man (OMIM)**, *http://www.ncbi.nlm.nih.gov/omim*
– **Picard**, *http://picard.sourceforge.net*
– **PLINK**, *http://pngu.mgh.harvard.edu/~purcell/plink*
– **PolyPhen-2**, *http://genetics.bwh.harvard.edu/pph2/dokuwiki/_media/hg0720.pdf*
– **PhyloP**, *http://compgen.bscb.cornell.edu/~acs/dless.pdf?*
– **RefSeq coding exons**, *http://www.ncbi.nlm.nih.gov/RefSeq*
– **SAMtools**, *http://samtools.sourceforge.net*
– **SIFT**, *http://sift.jcvi.org*
– **Uniprot**, *http://www.uniprot.org/uniprot/P35348*
